# Demographic and clinical characteristics of children and adolescents with headache and/or dizziness and hemodynamic responses to head-up tilt test

**DOI:** 10.1186/s13052-025-01976-y

**Published:** 2025-05-28

**Authors:** Runmei Zou, Shuo Wang, Fang Li, Ping Liu, Donglei Liao, Liqun Liu, Jing Liu, Hong Cai, Yuwen Wang, Cheng Wang

**Affiliations:** 1 Department of Pediatric Cardiovasology, The Second Xiangya Hospital, Children’s Medical Center, Central South University, No.139 Renmin Middle Road, Changsha, Hunan 410011 China; 2 Department of Pediatrics, Xiangya Hospital, Central South University, Changsha, Hunan 410008 China; 3 Department of Pediatric Neurology, Children’s Medical Center, The Second Xiangya Hospital, Central South University, Changsha, Hunan 410011 China; 4 The Children’s Cardiothoracic Vascular Center, Chenzhou First People’s Hospital, Chenzhou, Hunan 423000 China

**Keywords:** Headache, Dizziness, Head-up Tilt test, Autonomic function, Children

## Abstract

**Background:**

Headache and dizziness are common symptoms in children and adolescents, but the causes of headache/dizziness in most pediatric patients are not clearly defined. We intended to investigate the demographic and clinical features of pediatric patients with headache and/or dizziness and responses to head-up tilt test (HUTT).

**Methods:**

The demographic data and medical records of children and adolescents, with a primary complaint of headache and/or dizziness and undergoing HUTT between January 2001 and June 2023, were retrospectively reviewed and analyzed. Children and adolescents with headache and/or dizziness secondary to fever, trauma, or other obvious etiology were excluded. For those with positive responses to HUTT, the symptom score and HUTT responses were collected after treatment at follow-up.

**Results:**

Among 2709 patients with unexplained headache and/or dizziness, 1080 (39.87%) cases presented positive responses to HUTT, whereas 1629 (60.13%) cases had negative responses. Among patients with positive responses, 930 (34.33%) cases presented a response of vasovagal syncope, 143 (5.28%) cases of postural orthostatic tachycardia syndrome, and 7 (0.26%) cases of orthostatic hypertension. Multivariate Logistic regression analysis demonstrated that females, older age, and low body mass index percentile increased the risk of being positive responses. At follow-up, HUTT results of 236 patients were collected, among which 131 (55.51%) cases turned negative HUTT responses and had headache/dizziness symptoms improved after treatment targeted autonomic dysfunction.

**Conclusion:**

HUTT can be applied to evaluate the autonomic function of children and adolescents with headache and/or dizziness and assess the treatment responses in those patients with autonomic dysfunction.

**Supplementary Information:**

The online version contains supplementary material available at 10.1186/s13052-025-01976-y.

## Background

Headache is a common neurological symptom in children and adolescents, which brings significant disability [1]. Globally, nearly 60% of children and adolescents experience significant headache [2]. The prevalence of primary headache ranged from 10 to 20% in school-age children, and was over 50% in those under twenty years old [2, 3]. The causes of headaches vary from primary to secondary headaches. The primary headache includes migraine, tension-type headache, trigeminal autonomic cephalalgias, and other primary headache disorders, whereas the secondary headache occurs closely related to disorders that are known to cause headache, such as fever, trauma, cranial or cervical vascular disorder, infection, and so on [4]. Dizziness is a general term that describes several sensations of an impending fall, floating, or disequilibrium and indicates a dysfunction in the vestibular, somatosensory, or visual systems [5].

Headache is responsible for 0.5–4.5% of all visits to the emergency room [6], while dizziness accounts for more than 3% of presentations to emergency departments or outpatient clinics [7]. However, most cases with headache or dizziness have benign origins and present normal cranial magnetic resonance imaging (MRI) and electroencephalogram (EEG), but complain of recurrent headache and/or dizziness and visit doctors frequently. These patients and their families are profoundly troubled. Determining the causes and selecting appropriate treatment for headaches and/or dizziness is crucial to help relieve the burden of family and society, and improve the patients’ quality of life.

Head-up tilt test (HUTT) is an important tool to assess autonomic function and is applied to diagnose orthostatic intolerance in children [8]. Studies indicated that autonomic pathways are involved in headache and dizziness, and HUTT can help confirm a diagnosis and assess the treatment response in patients with autonomic dysfunction [9, 10]. This study aims to investigate the demographic and clinical features of children and adolescents with headache and/or dizziness and responses to HUTT.

## Materials and methods

### Study population and data collection

Children and adolescents with a headache and/or dizziness as their primary complaint were hospitalized in the Syncope Ward, The Second Xiangya Hospital, Central South University between January 2001 and June 2023. The demographic data and medical records were retrospectively reviewed by two researchers. All patients were evaluated as per standard guidelines [4, 11]. Inclusion criteria: children and adolescents aged under 18 years old; headache and/or dizziness as the primary complaint; underwent HUTT; with complete clinical data. Patients with headache and/or dizziness due to fever, trauma, and/or other obvious etiology such as meningitis, dental conditions, sinusitis, vision problems, and other systemic diseases were excluded.

### HUTT protocol

The HUTT protocol was conducted according to the previous study [12]. The test was noninvasive and approved by the Ethics Committee of The Second Xiangya Hospital, Central South University [Ethical Audit No. Study 012 (2014)]. Written informed consent was signed by the patients’ guardians before HUTT. Subjects stayed still on the table for 10 min, and then basic heart rate (HR), blood pressure (BP), and electrocardiogram (ECG) were recorded. Subsequently, subjects were tilted at 60◦ head upward. HR, BP, and ECG were recorded continuously until either 45 min duration or development of syncope or intolerable near syncope symptoms. If syncope occurred, subjects were rapidly put in the supine position. If syncope or pre-syncope did not occur, tilted posture was maintained, subjects were sublingually medicated with nitroglycerin (4–6 µg/kg, maximum dosage ≤ 300 µg), and HR, BP, and ECG were recorded for 20 min or syncope or pre-syncope occurred.

### Definition of positive responses to HUTT

Positive responses to HUTT included vasovagal syncope (VVS), postural orthostatic tachycardia syndrome (POTS), and orthostatic hypertension (OHT). VVS was defined as the development of syncope or pre-syncope during HUTT accompanied by any of the following responses, hypotension: systolic BP ≤ 80 mmHg and/or diastolic BP ≤ 50 mmHg, or over 25% decrease in mean BP; bradycardia: HR < 75 bpm for children aged 5–6 years, < 65 bpm for those aged 6–8 years, and < 60 bpm for those older than 8 years; ECG showed sinus arrest, junctional escape rhythm; atrioventricular block and asystole ≥ 3 s. POTS was defined using the following criteria: the presence of orthostatic intolerance symptoms (e.g., fatigue, headache, lightheadedness, blurring of vision, palpitations, tremulousness, and even syncope) and an orthostatic HR increment ≥ 40 bpm compared with the supine position, or an absolute orthostatic HR ≥ 130 bpm for children aged 5–12 years old or ≥ 125 bpm for adolescents aged 12–18 years, with a BP decrease < 20/10 mmHg during the first 10 min of the HUTT. OHT was defined as (within 3 min of HUTT) orthostatic intolerance symptoms and an increase in systolic BP ≥ 20 mmHg, and/or diastolic BP increments ≥ 25 mmHg in children between 5 and 12 years old, ≥ 20 mmHg in adolescents between 12 and 18 years old, or upright BP ≥ 130/90 mmHg in children between 5 and 12 years old and ≥ 140/90 mmHg in adolescents between 12 and 18 years old without an obvious change in HR [12, 13].

### Treatment and follow-up

Patients with positive responses received health education (avoidance of triggers, early recognition of prodromal symptoms, increased intake of water (daily water intake of 30–50 mL/kg) and salt and careful avoidance of agents that lower BP including diuretics), and tilt training (standing against a wall with the united ankles 15 cm from the wall, 5 min/episode, twice a day, and gradually increased to 20–30 min/episode) [14]. In addition, patients with VVS responses took oral rehydration salts (ORS, 250 mL, twice a day), and patients with POTS and OHT took metoprolol (0.5-1.0 mg/(kg·d) was administered orally in two divided doses) for treatment [12, 13]. The median follow-up time was 1.5 months (ranging from 1 month to 20 months). At follow-up, demographic information, symptom, and the responses to HUTT were collected from the HUTT case report form. Symptom score was utilized to assess the frequency of headache/dizziness before and at follow-up [15]:0 point indicated no episode of headache/ dizziness; 1 point, 1 episode per month; 2 points, 2–4 episodes per month; 3 points, 2–7 episodes per week; 4 points, more than once per day. The decrease of symptom score was calculated as symptom score before follow-up minus symptom score at follow-up.

### Statistical analysis

Statistical analysis was performed by SPSS 24.0 (IBM Corp., Armonk, NY, United States). Continuous variables for data following normal distribution were expressed as mean ± SD and analyzed by Student *t*-tests, or one-way ANOVA with polynomial contrasts and post hoc LSD among groups. Continuous variables for data not following normal distribution were expressed as the median with interquartile range and Mann-Whitney *U* test was utilized for analysis. Categorical data were expressed by frequencies and percentages, and analyzed by *x*^*2*^ test or Fisher’s exact test. Multiple Logistic regression was applied to analyze the association between positive responses to HUTT and related factors. *P* value < 0.05 was considered to be a statistically significant difference.

## Results

Among 2709 cases of children and adolescents (aged 5–18 years, 1486 (54.85%) males) with headache and/or dizziness, 1080 (39.87%) cases presented positive responses to HUTT and were enrolled in the positive group, whereas 1629 (60.13%) cases had negative responses to HUTT and were enrolled in the negative group. There were significant differences in the sex ratio and the ratio of body mass index (BMI) percentile between the two groups. The proportion of females in the positive group was higher than that of the negative group (49.35% vs. 42.36%, *P*<0.001). The proportion of patients with BMI<5th percentile in the positive group was higher than that in the negative group (9.72% vs. 6.45%, *P* = 0.002). The two groups had no significant difference in history duration and HR (all *P*>0.05). Compared with patients with negative responses, patients with positive responses presented older age (11.22 ± 2.48 vs. 9.76 ± 2.71 years, *P*<0.001), higher basic SBP (107.69 ± 9.89 vs. 106.36 ± 10.38 mmHg, *P* = 0.001), and higher basic DBP (66.13 ± 7.39 vs. 65.32 ± 7.62 mmHg, *P* = 0.006) (Table 1).

**Table 1:** Demographic and clinical characteristics of children and adolescents with headache and/or Dizziness [mean ± SD, M(Q1-Q3), n(%)]

Variables	Negative group	Positive group	Total	*P* value
Case	1629 (60.13)	1080(39.87)	2709	
Age (years)	9.76 ± 2.71	11.22 ± 2.48	10.34 ± 2.72	<0.001
Sex				<0.001
Male	939 (57.64)	547 (50.65)	1486 (54.85)	
Female	690 (42.36)	533 (49.35)	1223 (45.15)	
BMI percentile				<0.001
<5th	105 (6.45)	105 (9.72)	210 (7.75)	0.002
5-85th	1194 (73.29)	837 (77.50)	2031 (74.97)	0.013
>85-95th	157 (9.64)	79 (7.31)	236 (8.71)	0.036
>95th	173 (10.62)	59 (5.46)	232 (8.56)	<0.001
History duration (month)	3 (1–12)	6 (1–12)	4 (1–12)	0.056
Basic SBP (mmHg)	106.36 ± 10.38	107.69 ± 9.89	106.86 ± 10.37	0.001
Basic DBP (mmHg)	65.32 ± 7.62	66.13 ± 7.39	65.64 ± 7.54	0.006
Basic HR (beats/min)	77.89 ± 12.06	77.20 ± 13.35	77.62 ± 12.60	0.167

SBP, systolic blood pressure; DBP, diastolic blood pressure; HR, heart rate

Among 1080 children with positive HUTT responses, 930 (34.33%) cases presented a response of VVS, 143 (5.28%) cases of POTS, and 7 (0.26%) cases of OHT. There was a significant difference in the sex ratio between VVS, POTS, and OHT groups, patients with OHT presented male dominance compared with those with VVS or POTS (*P* = 0.036, Table 2).

**Table 2:** Hemodynamic types in children and adolescents with positive responses to HUTT [mean ± SD, M(Q1-Q3), n(%)]

Variables	VVS	POTS	OHT	*P* value
Case	930 (34.33)	143 (5.28)	7 (0.26)	
Age (years)	11.28 ± 2.49	10.78 ± 2.43	11.85 ± 1.67	0.062
Sex				0.036
Male	459 (49.35)	82 (57.34)	6 (85.71)	
Female	471(50.65)	61 (42.66)	1 (14.29)	
BMI percentile				0.090
<5th	87 (9.35)	18 (12.59)	0	
5-85th	718 (77.20)	114 (79.72)	5 (71.43)	
>85-95th	69 (7.42)	8 (5.59)	2 (28.57)	
>95th	56(6.02)	3 (2.10)	0	
History duration (month)	6 (1–12)	3 (1–24)	2(1-2.5)	0.234
Basic SBP	107.57 ± 9.76	108.10 ± 10.32	114.86 ± 15.72	0.132
Basic DBP	66.17 ± 7.39	65.90 ± 7.52	66.00 ± 4.54	0.920
Basic HR	77.01 ± 12.87	78.87 ± 16.10	68.57 ± 10.44	0.069

HUTT, head-up tilt test; VVS, vasovagal syncope; POTS, postural tachycardia syndrome; OHT, orthostatic hypertension; SBP, systolic blood pressure; DBP, diastolic blood pressure; HR, heart rate

As shown in Table 3, multivariate Logistic regression analysis demonstrated that sex, age, and BMI percentile were independent factors related to positive responses to HUTT. In relative to male patients, the rate of positive responses to HUTT was increased by 34.6% in female patients (*P*<0.001). With one-year increase in age, the risk of positive responses was raised by 23.5% (*P*<0.001). Compared to patients with BMI<5th percentile, the risk of positive responses was decreased by 37.3%, 60.2% and 71.1% in patients with BMI between 5-85th percentile,>85-95th percentile, and>95th percentile (*P* = 0.002, *P*<0.001, *P*<0.001, respectively), suggesting low BMI percentile was associated with the risk of positive responses.

**Table 3:** Multivariate logistic regression analysis between positive response to HUTT and related factors

Variables	*P*	OR	OR 95% CI	
			lower	upper
Age (year)	<0.001	1.235	1.195	1.276
Sex	<0.001	1.346	1.145	1.583
BMI percentile				
<5th		1.000		
5-85th	0.002	0.627	0.465	0.845
>85-95th	<0.001	0.398	0.265	0.596
>95th	<0.001	0.289	0.189	0.442
basic SBP (mmHg)	0.250	1.006	0.996	1.017
basic DBP (mmHg)	0.942	0.999	0.986	1.013

HUTT, head-up tilt test; SBP, systolic blood pressure; DBP, diastolic blood pressure

Patients with positive responses to HUTT received health education and tilt training. Patients with VVS responses took oral rehydration salts, and patients with POTS and OHT took metoprolol. After a median follow-up time of 1.5 months (ranging from 1 month to 20 months), HUTT results of 236 patients were collected, among which 131 (55.51%) patients turned negative HUTT response. At follow-up, the median decrease of symptom score in all 236 patients was more than 1 point. Symptom scores decreased more in those who turned negative HUTT responses than those who still presented positive responses (median 2 vs. 1 point, *P*<0.001, Table 4). These results suggested that patients with positive responses to HUTT had autonomic dysfunction and treatments targeted autonomic dysfunction are effective for these patients.

**Table 4:** Follow-up of children and adolescents with headache and/or Dizziness with positive HUTT response [mean ± SD, M(Q1-Q3), n (%)]

Variables	Still positive	Turn negative	*P* value
Case	105(44.49)	131(55.51)	
Age(years)	10.96 ± 2.12	10.64 ± 2.34	0.267
Sex			0.966
Male	58(55.23)	72(54.96)	
Female	47(44.76)	59(45.04)	
Follow-up time (month)	1.5(1-5.5)	1.5(1-3.5)	0.527
Decrease of symptom score	1(0–1)	2(1–2)	<0.001

HUTT, head-up tilt test; SBP, systolic blood pressure; DBP, diastolic blood pressure

## Discussion

In the present study, we found that 39.87% of children and adolescents with headache/dizziness had positive responses to HUTT. Females, older age, and low BMI percentile increased the risk of being positive responses. For those patients with positive responses to HUTT, more than half of these patients turned negative responses to HUTT at follow-up. They also presented more decreased symptom scores compared with those who still presented positive responses, suggesting improvement of headache/dizziness symptoms in these patients.

Headache and dizziness are the most frequent neurological symptoms in childhood, and have an important adverse impact on children and their families [16]. Headache is associated with many factors, such as genetics, hormones, stress, dehydration, diet, and medication [17]. Additionally, autonomic dysfunction is involved in the pathogenesis of multiple primary and secondary headache disorders [9]. The central autonomic network, as well as peripheral sympathetic and parasympathetic efferents, is thought to be responsible for generating headache and cranial autonomic symptoms [18]. In our study, secondary headache due to fever, trauma, and/or other obvious etiology such as meningitis, dental conditions, and sinusitis was excluded, but these patients complained of recurrent headache/dizziness and visited doctors frequently. Recurrent headache/ dizziness substantially impacts health status, school-related activities, sports and social activities, and quality of life [17, 19].

HUTT, an important tool to assess cardiovascular autonomic function [20, 21], can induce a series of autonomic dysfunction symptoms such as chest tightness, and dizziness. Previous studies demonstrated that HUTT can be utilized to diagnose the hemodynamic pattern of unexplained sighing and chest pain in children and adolescents [22, 23]. In our study, nearly 40% of patients with headache and/or dizziness had positive responses to HUTT, including hemodynamic types of VVS, POTS, and OHT, suggesting the existence of cardiovascular autonomic dysfunction in these patients. VVS, caused by an abrupt decrease of cerebral blood flow with transient loss of consciousness due to bradycardia and hypotension, is closely associated with headache [24]. Approximately 22% of patients with dizziness can be attributed to VVS [7]. In our study, nearly 35% of patients with headache/dizziness had a response of VVS. Headache was the most common comorbidity in pediatric patients with POTS [25]. 46% of patients with orthostatic intolerance and abnormal HUTT presented symptom of headache [26]. Dizziness and headache were the most frequent symptoms in children with POTS [27]. In our study, 5.31% of patients with headache/dizziness presented a response of POTS during HUTT. These results suggested that headache/dizziness and POTS are closely correlated. High prevalence of comorbidity of headache/dizziness and POTS may be due to dysregulation of the brainstem including the raphe nuclei or the locus coeruleus.

Logistic regression analysis was performed to investigate the risk factors related to positive responses to HUTT, and sex, age, and BMI percentile were found to be independent factors associated with positive responses. A previous study demonstrated that females are more likely to have headaches than males [2]. In our study, more males have headaches and/or dizziness but the proportion of females was higher in the positive group than that in the negative group as autonomic dysfunction is more common in females. In healthy subjects, age and sex had a significant influence on plasma norepinephrine concentration at baseline and in the upright position, which modulates cardiovascular and neuroendocrine responses to orthostatic challenge [28]. Due to less splanchnic vasoconstriction during HUTT, females tend to have lower tilt-table tolerance than males. When tilting, females are more likely to present positive responses [29].

Other than sex, age and BMI percentile were independent effects on the responses to HUTT in our study. Cardiac baroreflex latency increases while the degree of cardiac baroreflex decreases with age [30]. Older patients tend to have positive responses during active standing, which is consistent with the results of our study. During HUTT, the head is lifted above the heart, resulting in gravitational pooling in the lower extremities [31], subsequently following a decrease of venous return and cardiac output. The taller the patient is, the more volume accumulates in the lower extremities, leading to the tendency of positive responses to HUTT. A previous study demonstrated that a lower BMI was associated with hypovolemia in POTS patients [32]. HUTT-positive patients showed a significantly lower BMI compared to HUTT-negative patients [33]. In our study, the proportion of patients with BMI<5th percentile in the positive group was higher than that in the negative group. Compared to patients with BMI<5th percentile, the risk of being positive responses was decreased in patients with BMI>5th percentile. These results suggested that patients with lower BMI are more likely to be positive responses to HUTT.

For those with positive HUTT responses, treatment strategies including education, tilt training, ORS or metoprolol are effective in improving the responses to HUTT and reducing the frequency of headache/dizziness during the follow-up, suggesting the improvement of autonomic function.

There are some limitations in the study. This was a retrospective and single-center study, which may result in bias and underestimation of some important factors. Most of the patients have been lost at follow-up, which resulted in a small sample of patients re-performing HUTT.

## Conclusion

In conclusion, HUTT can be applied to evaluate the autonomic function of children and adolescents with headache and/or dizziness and assess the treatment responses in those patients with autonomic dysfunction.

## Electronic supplementary material

Below is the link to the electronic supplementary material.

Supplementary Material 1

## Data Availability

The original contributions presented in the study are included in the article/supplementary material, further inquiries can be directed to the corresponding author.

## Abbreviations

HUTT: Head-up tilt test
MRI: Magnetic resonance imaging
ECG: Electrocardiogram
EEG: Electroencephalogram
HR: Heart rate
BP: Blood pressure
VVS: Vasovagal syncope
POTS: Postural orthostatic tachycardia syndrome
OHT: Orthostatic hypertension
ORS: Oral rehydration salts
BMI: Body mass index
