# Supplementary material for: Demographic and clinical characteristics of children and adolescents with headache and/or dizziness and hemodynamic responses to head-up tilt test
Source: Ital J Pediatr. 2025 May 28;51:161. doi: 10.1186/s13052-025-01976-y (PMC12121244; doi:10.1186/s13052-025-01976-y)
Supplement: Supplementary file 1 — Supplementary Material 1 [file 13052_2025_1976_MOESM1_ESM.doc]

**Cover Letter**

Dear editorial board of **Ital J Pediatr**,

We would like to submit the enclosed manuscript entitled “Demographic and clinical characteristics of children and adolescents with headache and/or dizziness and hemodynamic responses to head-up tilt test”, by Runmei Zou, Shuo Wang, Fang Li, Ping Liu, Donglei Liao, Liqun Liu, Jing Liu, Hong Cai, Yuwen Wang and Cheng Wang, which we wish to considered for publication in **Ital J Pediatr**. We would like to declare that there are no prior publications or submissions with any overlapping information, including studies and patients. The manuscript has not been and will not be submitted to any other journal while it is under consideration by **Ital J Pediatr**. No potential conflict of interest exits in the submission of this manuscript.

No honorarium, grant, or other form of payment was given to anyone to produce the manuscript. Each author listed on the manuscript has seen and approved the submission of this version of the manuscript and takes full responsibility for the manuscript.

We deeply appreciate your consideration of our manuscript, and are looking forward to receiving comments from the reviewers. If you have any queries, please don’t hesitate to contact me at the address below.

Thank you and the best regards.

Yours sincerely,

Cheng Wang on behalf of the authors.

**Corresponding authors**: Prof. Cheng Wang

**E-mail**: [wangcheng2nd@csu.edu.cn](mailto:wangcheng2nd@csu.edu.cn)

**Tel:** 86 731 852 95258

**ORCID:** 0000-0002-7120-0654

Department of Pediatric Cardiovasology, Children's Medical Center, The Second Xiangya Hospital, Central South University, Changsha, Hunan, 410011, China.
